# Impact of uncertainty quantification through conformal prediction on volume assessment from deep learning-based MRI prostate segmentation

**DOI:** 10.1186/s13244-024-01863-w

**Published:** 2024-11-29

**Authors:** Marius Gade, Kevin Mekhaphan Nguyen, Sol Gedde, Alvaro Fernandez-Quilez

**Affiliations:** 1 Department of Electrical Engineering and Computer Science, University of Stavanger, Stavanger, Norway; 2 Stavanger Medical Imaging Laboratory (SMIL), Department of Radiology, Stavanger University Hospital, Stavanger, Norway

**Keywords:** Magnetic resonance imaging, Deep learning, Prostate, Conformal prediction, Uncertainty

## Abstract

**Objectives:**

To estimate the uncertainty of a deep learning (DL)-based prostate segmentation algorithm through conformal prediction (CP) and to assess its effect on the calculation of the prostate volume (PV) in patients at risk of prostate cancer (PC).

**Methods:**

Three-hundred seventy-seven multi-center 3-Tesla axial T2-weighted exams from biopsied males (66.64 $\pm$ 7.47 years) at risk of PC were retrospectively included in the study. Assessment of PV based on PI-RADS 2.1 ellipsoid formula (${{{\rm{PV}}}}_{{ref}}$) was available for included patients. Prostate segmentations were obtained from a DL model and used to calculate the PV (${{{\rm{PV}}}}_{{DL}}$). CP was applied at a confidence level of 85% to flag unreliable pixel segmentations of the DL model. Subsequently, the PV (${{{\rm{PV}}}}_{{CP}}$) was calculated when disregarding uncertain pixel segmentations. Agreement between ${{{\rm{PV}}}}_{{DL}}$ and ${{{\rm{PV}}}}_{{CP}}$ was evaluated against the reference standard ${{{\rm{PV}}}}_{{ref}}$. Intraclass correlation coefficient (ICC) and Bland–Altman plots were used to assess the agreement. The relative volume difference (RVD) was used to evaluate the PV calculation accuracy, and the Wilcoxon Signed-Rank Test was used to assess statistical differences. A *p*-value < 0.05 was considered statistically significant.

**Results:**

Conformal prediction significantly reduced RVD when compared to the DL algorithm (RVD = − 2.81 $\pm$ 8.85 and RVD = −8.01 $\pm$ 11.50). ${{{\rm{PV}}}}_{{CP}}$ showed a significantly larger agreement than ${{{\rm{PV}}}}_{{DL}}$ when using the reference standard ${{{\rm{PV}}}}_{{ref}}$ (mean difference (95% limits of agreement) ${{{\rm{PV}}}}_{{CP}}$: 1.27 mL (− 13.64; 16.17 mL) ${{{\rm{PV}}}}_{{DL}}$: 6.07 mL (− 14.29; 26.42 mL)), with an excellent ICC (${{{\rm{PV}}}}_{{CP}}$: 0.97 (95% CI: 0.97 to 0.98)).

**Conclusion:**

Uncertainty quantification through CP increases the accuracy and reliability of DL-based PV assessment in patients at risk of PC.

**Critical relevance statement:**

Conformal prediction can flag uncertain pixel predictions of DL-based prostate MRI segmentation at a desired confidence level, increasing the reliability and safety of prostate volume assessment in patients at risk of prostate cancer.

**Key Points:**

- Conformal prediction can flag uncertain pixel predictions of prostate segmentations at a user-defined confidence level.
- Deep learning with conformal prediction shows high accuracy in prostate volumetric assessment.
- Agreement between automatic and ellipsoid-derived volume was significantly larger with conformal prediction.

**Graphical Abstract:**

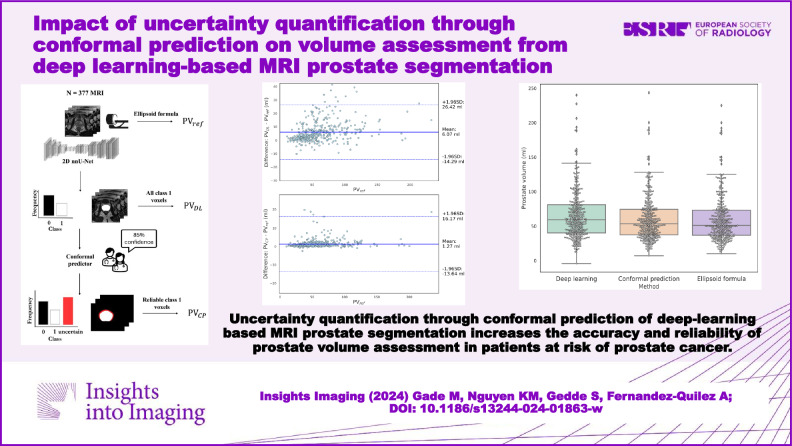

## Introduction

Prostate cancer (PC) is the second most common cancer in men worldwide [1]. PC diagnosis relies on an initial assessment of prostate-specific antigen (PSA) levels [2]. Dividing the PSA by the prostate volume (PV) yields the PSA density (PSAd), an important PC biomarker for biopsy referral [3]. In this regard, magnetic resonance imaging (MRI) has become integral in recent years for determining PV because of its resolution and ability to extract in vivo functional information [4–6].

According to the Prostate Imaging Reporting & Data System (PI-RADS) guidelines recommendations, PV should be calculated using the ellipsoid formula (EF) [7, 8]. The formula relies on the manual measurement of the prostate’s depth, height, and width by the radiologist. Although relatively accurate, its use is time-consuming and prone to inter-reader variability, and its underlying assumptions about the ellipsoid shape pose challenges in complex region anatomies such as the apex [8–10]. Manual contouring using external software [8, 11, 12] is the most accurate alternative, but is time-consuming and exhibits variability, even among experts [4, 10, 11].

In recent years, deep-learning (DL) algorithms have shown promise in automatically segmenting the prostate and assessing the PV [4, 8]. Variants of the U-Net model obtained through the nnU-Net framework are popular choices [4, 13, 14]. However, the increase in DL alternatives for PV calculation has not translated into clinical adoption [15, 16]. Barriers include concerns about their reliability and their tendency to produce overconfident predictions, leading to underperformance in clinical contexts compared to their in-silico results [15, 17].

To increase the reliability of DL for PV assessment, it is crucial to estimate the uncertainty associated with each pixel prediction of the prostate segmentation [16, 18]. Conformal prediction (CP) is a standard method for uncertainty quantification (UQ) that provides statistically valid measures of confidence in a distribution free and model-agnostic manner [19, 20]. With CP, the physician can set a desired confidence level (e.g., 85%), such that CP will return a region around the original prediction that contains the true label with that probability. Predictions below this confidence level are flagged for human intervention, ensuring that only reliable predictions are employed for PV calculation.

Among CP methodologies, Mondrian inductive conformal prediction (ICP) is particularly effective for addressing pixel-level uncertainties [21, 22]. Mondrian ICP can generate robust uncertainty estimates without the significant computational overhead associated with alternatives like Venn predictors or cross-conformal prediction [21, 22]. Other UQ methods outside the CP family, such as Monte Carlo dropout or ensemble methods, also present their own challenges, including reliance on specific assumptions about output distributions and high computational demands [22].

While other studies have explored DL for prostate segmentation, to our knowledge, none have investigated the impact of UQ in PV assessment from DL-based prostate segmentation. Furthermore, despite the growing popularity of CP in recent years [20, 23], to date, no DL-based prostate segmentation has employed Mondrian ICP for UQ. Against this background, we aimed to investigate the effect of Mondrian ICP on UQ in DL-based PV assessment. We hypothesized that Mondrian ICP can significantly improve the accuracy of DL-based PV assessment by enabling the use of reliable pixel predictions with a pre-defined level of certainty from the DL segmentation model.

## Materials and methods

The data for this retrospective, multi-center study was approved by the institutional or regional review board of all contributing centers. Informed consent was waived given the retrospective, scientific and de-identified use of the data [24].

### Patient cohort

Prostate imaging-cancer artificial intelligence (PI-CAI) is a multi-center and multi-scanner collection of prostate MRI exams aimed at validating DL algorithms. Patient exams were collected based on suspected PC due to elevated PSA levels and abnormal digital rectal examination (DRE) findings. Patients included in PI-CAI had no history of previous treatment or biopsy-confirmed PC findings [24].

The publicly available PI-CAI dataset consists of 1,500 T2-weighted (T2w) and diffusion-weighted (DW) MRI exams collected between January 2012 and December 2021 from four centers and two MRI vendors. Clinical variables including PSA levels (ng/mL), prostate volume (mL), PSAd (ng/mL^2^), Gleason scores and patient age (years) were available when reported during clinical routine [22]. Reported PVs were calculated following the EF formula suggested by the PI-RADS 2.1 guidelines [7, 8].

Out of the available data, 1001 exams had prostate segmentations at the time of the study. From these, 204 were annotated by two radiologist residents and two board-certified radiologists and were part of the ProstateX publicly available dataset [25]. The remaining 797 exams had segmentations generated by a DL algorithm supervised by an expert radiologist with more than 7 years of experience [2, 24]. All human-supervised annotations were performed with the open-source ITK-SNAP v3.80 software (http://www.itksnap.org/).

**Fig. 1:**
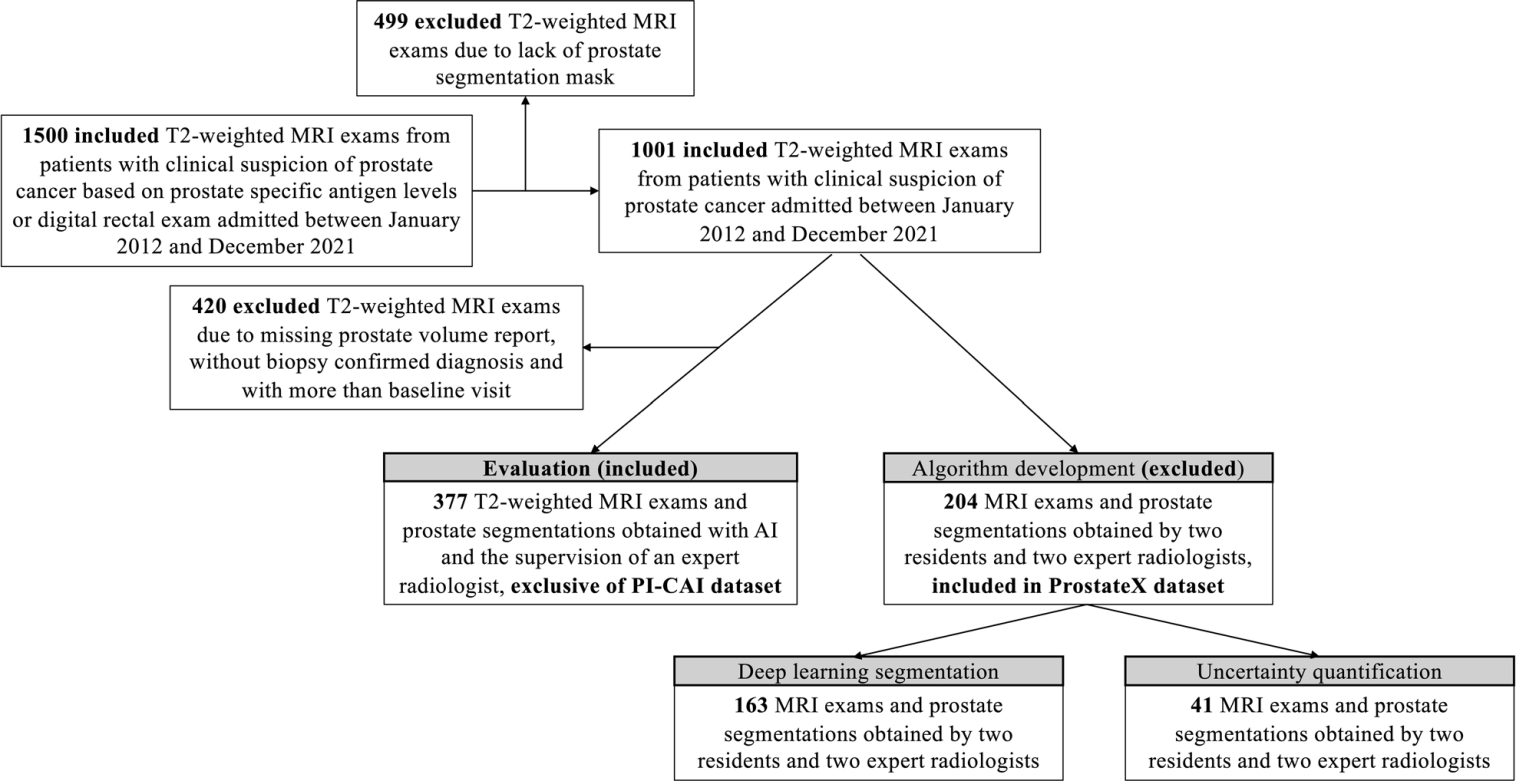
Patient inclusion criteria and resulting MRI exams used for algorithm development and for prostate volume evaluation

As shown in Fig. 1, 377 patients met the study inclusion criteria: (1) Biopsy-confirmed diagnosis, (2) Available prostate segmentation, (3) Reported PL during clinical routine, (4) No overlap with the 204 ProstateX patients [6, 25] previously used for the development of the prostate segmentation DL model and conformal predictor used in the work.

### MRI and pre-processing

We used axial T2-weighted (T2w) with available prostate segmentations. Images were acquired with various commercial 1.5 Tesla (T) and 3.0 T scanners (Siemens Healthineers, Erlangen, Germany; Philips Medical Systems, Eindhoven, the Netherlands), with heterogeneous in-plane resolution (median: 0.5 mm range: 0.2–0.8 mm), slice thickness (median: 3.5 mm range: 2.2–5.0 mm) and with a surface coil [24].

To palliate the visual differences from multi-center and multi-scanner acquisitions, we normalized the pixel intensity and re-sampled to a common space of reference of 0.5 mm × 0.5 mm × 3.0 mm with third-order spline interpolation [13]. For the prostate segmentation masks, spline interpolation was replaced with linear interpolation [13].

### Deep learning for automatic prostate segmentation

Automatic prostate segmentation utilized a previously developed 2D nnU-Net, leveraging the self-configuring nnU-Net framework [13]. The choice was made based on reported results in prostate segmentation tasks. A detailed description of the nnU-Net framework and training of the model can be found elsewhere [6, 13].

Briefly, the 2D nnU-Net model was developed using 163 patients (median age: 66 range: 48-83 years) with axial T2w MRI exams and prostate segmentations. The cohort was obtained by splitting the 204 ProstateX patients into a training set and a calibration set (Fig. 1) [25]. The cohort was exclusively used for model development and excluded in the evaluation of this work (Fig. 1). The model for automatic prostate segmentation is an ensemble of five distinct 2D nnU-Net models. Each of these models was generated through a five-fold cross-validation process, resulting in five separate models trained on different subsets of the training data. During testing, the segmentation masks from these five models were averaged to create the final segmentation mask [5]. Every individual model was trained for 300 epochs and implemented with TensorFlow (version 2.9.17) and Python (version 3.9.12; Python Software Foundation, Wilmington, DE, USA) on a single NVIDIA A100 GPU with 40GB RAM (NVIDIA Corporation, Santa Clara, California, USA).

### Conformal prediction for prostate segmentation uncertainty quantification

**Fig. 2:**
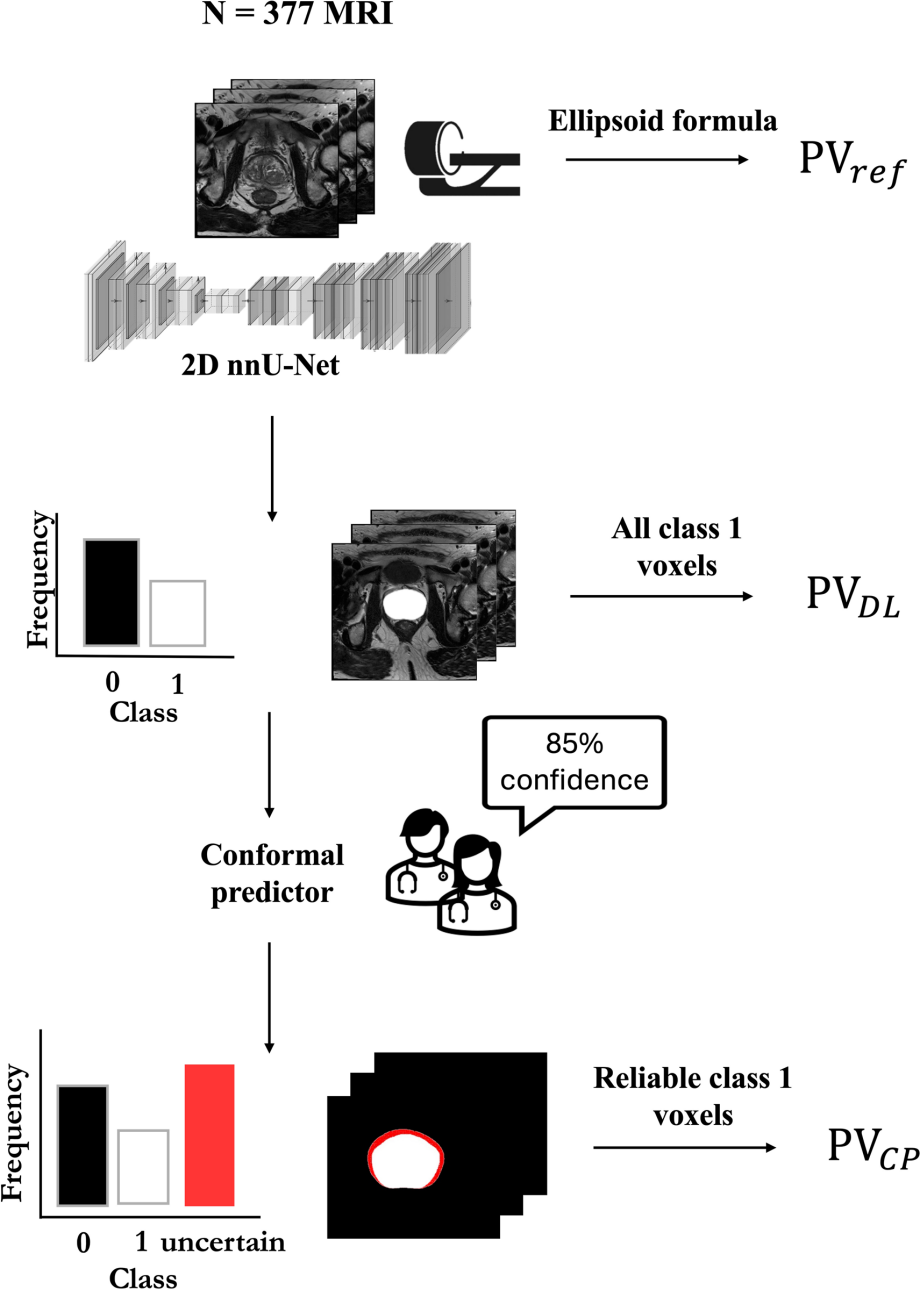
The deep-learning segmentation system delivers a per-pixel probability of a pixel belonging to the class label background {0} or prostate {1}. The output of the system is used as the input for the conformal predictor, which identifies unreliable pixel predictions based on a pre-specified 85% confidence level. Finally, we measure the agreement between the prostate volumes obtained from the deep-learning system ${{{\rm{PV}}}}_{{DL}}$, from the conformal predictor ${{{\rm{PV}}}}_{{CP}}$ obtained by disregarding unreliable pixel predictions and ${{{\rm{PV}}}}_{{ref}}$ calculated through the PI-RADS 2.1 ellipsoid formula by an experienced radiologist

We interpret the DL segmentation model as a collection of binary classifiers, providing per-pixel probabilities of each pixel belonging to the class label {0}: background or {1}: prostate. The CP framework is naturally designed to work with the output probabilities of the DL model. By taking the DL model pixel-wise probabilities as input, our CP framework is able to quantify the uncertainty at the pixel-level. This, in turn, influences global measures from the segmentation, such as PV. The application of the CP framework to the DL prostate segmentation model is depicted in Fig. 2.

We constructed our conformal predictor leveraging a Mondrian inductive conformal classifier [19], which guarantees a desired error rate for each class (i.e. background and prostate). The predictor takes as input pixel predicted probabilities by the DL model for a given T2w MRI exam, and outputs a predicted region (set of class labels) for each pixel. This contrasts with the single-value pixel prediction from the original DL segmentation model. Labels are only included in the pixel-wise predicted region if the DL model confidence in that label is higher than the user-specified confidence level (Fig. 2). For instance, in the DL-based segmentation models each pixel can only be classified as {0}: background or {1}: prostate. However, the Mondrian CP can output {0}: background, {1}: prostate, {0,1}: uncertain or {0,0} uncertain (empty). A prediction is flagged as uncertain on the basis that the model cannot assign a unique class for a given confidence level. To illustrate, for a T2w MRI exam with dimensions 320 × 320 × 23, the Mondrian predictor would receive 320 × 320 × 23 pixel predicted probabilities, a user-specified confidence level (e.g., 90%) and would output 320 × 320 × 23 pixel-wise predicted regions based on that confidence level (Fig. 2).

To train the Mondrian conformal classifier, we use the calibration set of 41 patients’ axial T2w MRI exams and prostate segmentations (Fig. 1), which are part of the 204 ProstateX patients excluded from this work [25]. The CP model was implemented with TensorFlow (version 2.9.17) using Python (version 3.9.12; Python Software Foundation, Wilmington, DE, USA) on a single NVIDIA A100 GPU with 40GB RAM (NVIDIA Corporation, Santa Clara, California, USA).

### Automatic prostate volume assessment

To assess the effect of CP on the PV calculation, we compared PV calculations obtained from the DL-based prostate segmentation without CP (${{{\rm{PV}}}}_{{DL}}$) and with CP (${{{\rm{PV}}}}_{{CP}}$). We compare also the segmentation quality of the DL model with and without CP, given its influence on the PV calculation. Based on the intended use of CP, we disregard pixel predictions flagged as uncertain in the calculations. The choice is intended to simulate a hypothetical clinical deployment scenario, where unreliable predictions would be disregarded until further intervention by a physician or specialist (Fig. 2). For the primary evaluation, we choose a confidence level of 85% corresponding to an alpha of 0.15 based on previously radiologist inter-observer reported agreements for T2w prostate segmentation [10, 14]. In addition, we conducted experiments across a range of alpha values (0.01, 0.05, 0.10, and 0.20) to assess the effect of the different confidence levels that fall above and below human expert consensus.

We assess quantitatively CP’s effect on PV calculation using the relative volume difference (RVD) between the calculated PV from a given segmented T2w MRI exam and the reference volume obtained with the EF formula (${{{\rm{PV}}}}_{{ref}}$). RVD values can range from –1 to +1, indicating underestimation or overestimation of the ${{{\rm{PV}}}}_{{ref}}$, respectively [14]. We leverage the dice score coefficient (DSC) and average surface distance (ASD) to evaluate the segmentation performance [11, 26]. The DSC values can range from 0 to +1, with +1 indicating total overlap, whilst ASD is calculated as the average distance between pixels in the reference and the predicted segmentations, where values closer to 0 indicate greater similarity between the surfaces [11, 25].

### Statistical analysis

Continuous variables statistics were reported as mean $\pm \,$SD (normally distributed) and median (interquartile range Q1–Q3) (non-normally distributed). Categorical variables were reported as counts and percentages (*N* (%)).

We assessed differences between DSC, ASD and RVD from the DL algorithm without CP and with CP. The expected calibration error (ECE) and Brier score (BS) were also assessed as a measure of the calibration of the methods. Wilcoxon Signed-Rank tests and paired Student’s t-test were employed to assess statistical differences based on normality.

Agreement between ${{{\rm{PV}}}}_{{DL}}$, ${{{\rm{PV}}}}_{{CP}},$ and ${{{\rm{PV}}}}_{{ref}}$ was evaluated using Bland–Altman plots and intraclass correlation coefficient (ICC), categorized as poor, moderate, good, or excellent for ICC < 0.50, 0.50 to 0.75, 0.75 to 0.90, and > 0.90 [8]. We first compared ${{{\rm{PV}}}}_{{DL}}$ and the ellipsoid-determined volume ${{{\rm{PV}}}}_{{ref}}$ followed by ${{{\rm{PV}}}}_{{CP}}$ with ${{{\rm{PV}}}}_{{ref}}$. Spearman correlation analyzed the agreement with the reference standard ${{{\rm{PV}}}}_{{ref}}$. Inter-reader agreement between automatically derived ${{{\rm{PV}}}}_{{DL}}$ and ${{{\rm{PV}}}}_{{CP}}$ measures was assessed through Bland–Altman plots, and a boxplot was used to present the distribution of volumes according to the different methods.

All analyses were performed in Python 3 (https://www.python.org/downloads/) with the open-sourced statsmodels 0.14.0 module (https://www.statsmodels.org/stable/index.html). A *p*-value < 0.05 was considered statistically significant.

## Results

The included cohort consisted of 377 patients at risk of PC (66.64 $\pm$ 7.47 years). Table 1 depicts the demographic and clinical characteristics of the cohort. Included patients had a median PSA of 8.80 ng/mL (6.00–12.80) and an average ${{{\rm{PV}}}}_{{ref}}$ of 59.43 mL (59.43 $\pm$ 32.47 mL). The majority of patients had a ${{{\rm{PV}}}}_{{ref}}$ larger than 50 mL (54.91%).

**Table 1:** Demographic and clinical characteristics of study participants

Characteristic	Patients (*N* = 377)
Age (years)	66.64 $\pm$ 7.47
PSA (ng/mL)	8.80 (6.00 and 12.80)
Prostate volume (mL)	59.43 $\pm$ 32.47
$\le \,$ 35 mL	77 (20.42)
$> \,$ 35 mL and $< \,$ 50 mL	93 (24.67)
$\ge \,$ 50 mL	207 (54.91)
PSAd (ng/mL^2^)	0.17 (0.11 and 0.25)
Biopsy type	
Systematic	118 (31.30%)
MRI guided	144 (38.20%)
MRI (+ systematic)	105 (27.85%)
Radical prostatectomy	10 (2.65%)
Biopsy results	
Negative	138 (36.60)
Gleason score $< \,$ 7	94 (24.94)
Gleason score $\ge$ 7	145 (38.46)

*PSA* prostate-specific antigen, *PSAd* prostate-specific antigen density

### Prostate segmentation accuracy

As shown in Table 2, the 2D nnU-Net DL model achieved an average DSC of 80.57 $\pm$ 8.96%, a median ASD of 0.75 mm (0.54–1.56 mm), and an average RVD of 8.01 $\pm$ 11.50%. When applying CP with an alpha of 0.15, the average DSC significantly increased to 93.81 $\pm$ 7.37%, the median ASD significantly decreased to 0.10 mm (0.04–0.21 mm), and the average RVD significantly decreased to 2.81 $\pm$ 8.85%. Additionally, the metrics were stratified by volume categories [3] (small: $\le$ 35 mL, medium: $>$ 35 mL and $\le$ 50 mL, and medium-large: $>$ 50 mL), as detailed in Appendix E1 Table S1. The results indicate that the performance of CP with an alpha of 0.15 remained consistent across the volume categories.

**Table 2:** Effect of uncertainty quantification through conformal prediction in deep-learning-based prostate volume assessment and prostate segmentation. Higher values for DSC are better, whilst values closer to 0 for RVD and ASD are better. Conformal prediction was applied with a confidence level of 85%

	PV and segmentation assessment (*N* = 377)		*p*-value
Value	DL algorithm without CP	DL algorithm with CP	
DSC (%)	80.57 $\pm$ 8.96	93.81 $\pm$ 7.37	**<** **0.001**^**a**^
ASD (mm)	0.75 (0.54 and 1.56)	0.10 (0.04 and 0.21)	**<** **0.001**^**a**^
RVD (%)	8.01 $\pm$ 11.50	2.81 $\pm$ 8.85	**<** **0.001**^**a**^
PV (mL)	65.50 $\pm$ 35.09	60.72 $\pm$ 33.06	**<** **0.001**^**a**^

*DL* Deep learning, *CP* Conformal prediction, *DSC* Dice score coefficient, *ASD* average surface distance, *RVD* relative volume difference, *PV* prostate volume

^a^ *p*-values < 0.05 were considered statistically significant, and presented as bold

Figures S1 and  S2 included in Appendix E1 present the results of the effect of various alpha values below human expert consensus (0.01, 0.05, 0.10) and above it (0.20). These results indicate that lower and higher alpha values led to under and over-segmentation, respectively, with decreased DSC, increased ASD, and increased RVD when compared to an alpha value of 0.15. In all cases, the metrics showed a significant improvement when compared to the 2D nnU-Net DL without CP.

**Fig. 3:**
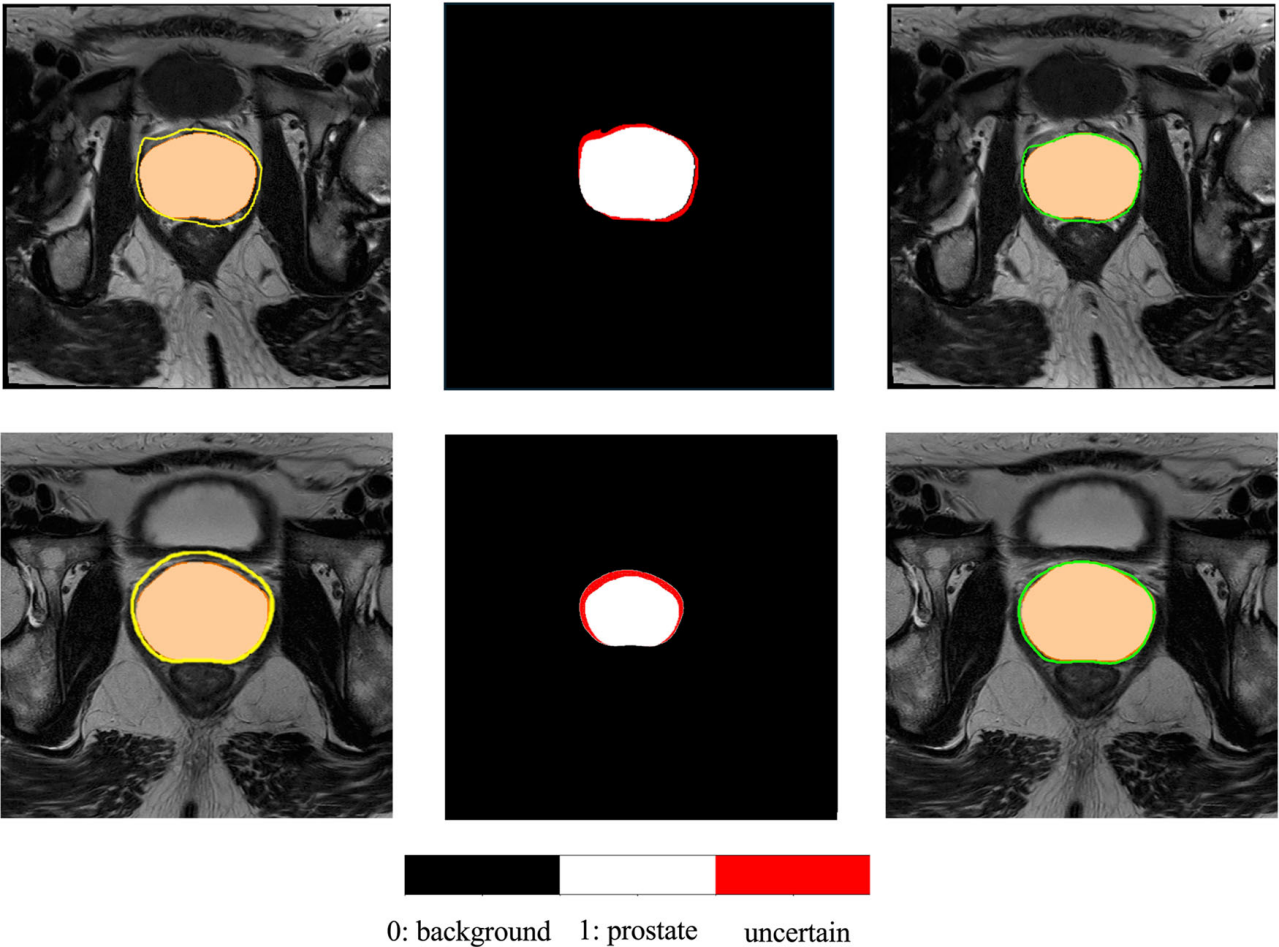
In each row, resulting segmentations for one slice from two different T2w patient MRI exams. The left column is outlined prostate contour resulting from the deep-learning algorithm (yellow), and in the middle is the result of applying the conformal prediction to the output of the deep-learning algorithm. In the right column, outlined prostate contour resulting from withholding pixel predictions flagged as unreliable by the conformal predictor. Conformal prediction was applied with a confidence level of 85%

As depicted in Table 3, both the average ECE and BS were significantly lower when applying CP with an alpha of 0.15 (ECE: 0.13 $\pm$ 0.06%; BS: 0.05 $\pm$ 0.10%). Additionally, the calibration metrics were stratified by volume categories [3] (small: $\le$ 35 mL, medium: $>$ 35 mL and $\le$ 50 mL, and medium-large: $>$ 50 mL), as detailed in Appendix E1 Table S2. The results indicate that the improvement in calibration when applying CP with an alpha of 0.15 remained consistent across the volume categories. A qualitative example of the effect of CP on the segmentations obtained by the DL model is shown in Fig. 3, where the segmentation accuracy improves after disregarding uncertain pixels flagged by CP.

**Table 3:** Effect of uncertainty quantification through conformal prediction in the calibration of the deep learning-based prostate segmentation. Lower values for ECE and BS are better. Conformal prediction was applied with a confidence level of 85%

	Calibration (*N* = 377)		*p*-value
Value	DL algorithm without CP	DL algorithm with CP	
ECE (%)	0.79 $\pm$ 0.14	0.13 $\pm$ 0.06	**<** **0.001**^**a**^
BS (%)	0.28 $\pm$ 0.20	0.05 $\pm$ 0.10	**<** **0.001**^**a**^

*ECE* expected calibration error, *BS* Brier score

^a^ *p*-values < 0.05 were considered statistically significant, and presented as bold

### Prostate volume characterization

**Fig. 4:**
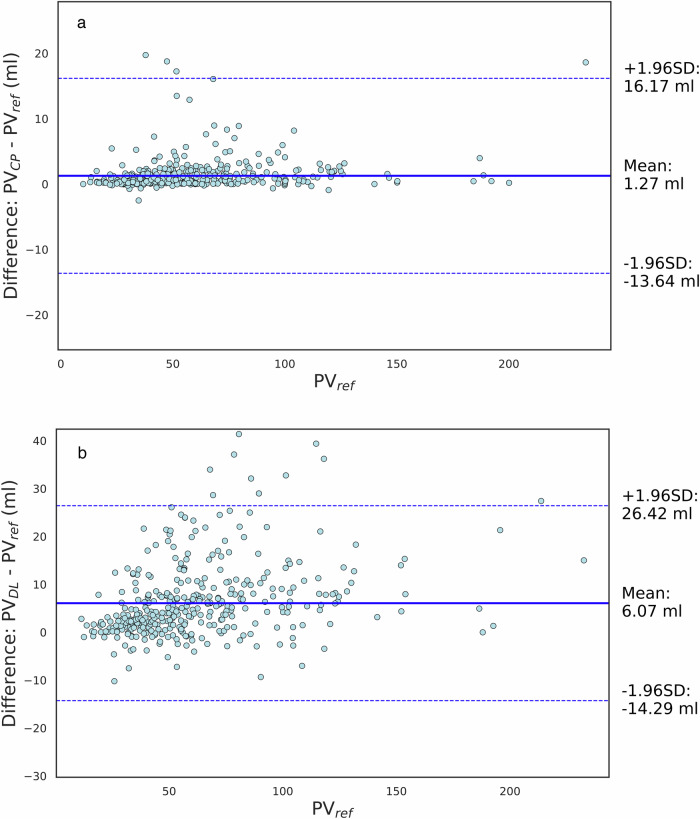
**a** Bland–Altman plot comparing the prostate volume obtained from applying conformal predictor ${{{\rm{PV}}}}_{{CP}}$ with the volume resulting from applying the ellipsoid formula ${{{\rm{PV}}}}_{{ref}}$. **b** Bland–Altman plot comparing the prostate volume calculation resulting from the deep-learning algorithm ${{{\rm{PV}}}}_{{DL}}$ with the volume resulting from applying the ellipsoid formula ${{{\rm{PV}}}}_{{ref}}$. The solid lines represent the mean difference and the dashed lines the limits of the agreements, calculated as a mean difference $\pm$  1.96 SD. Conformal prediction was applied with a confidence level of 85%

Agreement analysis using ${{{\rm{PV}}}}_{{ref}}$ as the reference standard and with alpha 0.15 is presented as a Bland–Altman plot in Fig. 4. The mean difference and corresponding 95% limits of agreement were significantly lower and narrower for ${{{\rm{PV}}}}_{{CP}}$ (Fig. 4a) than for ${{{\rm{PV}}}}_{{DL}}$ (Fig. 4b) (mean difference (95% limits of agreement) ${{{\rm{PV}}}}_{{CP}}$: 1.27 mL (− 13.64; 16.17 mL) ${{{\rm{PV}}}}_{{DL}}$: 6.07 mL (−14.29; 26.42 mL)). As shown in the Bland–Altman plot, there was an overall tendency of the DL model to overestimate PVs to a larger extent than when applying CP. The ICC (mean (95% CI)) was significantly larger for ${{{\rm{PV}}}}_{{CP}}$ (0.97 (0.97; 0.98)) than for ${{{\rm{PV}}}}_{{DL}}$ (0.94 (0.94; 0.96)). Both measures presented an excellent agreement with ${{{\rm{PV}}}}_{{ref}}$.

**Fig. 5:**
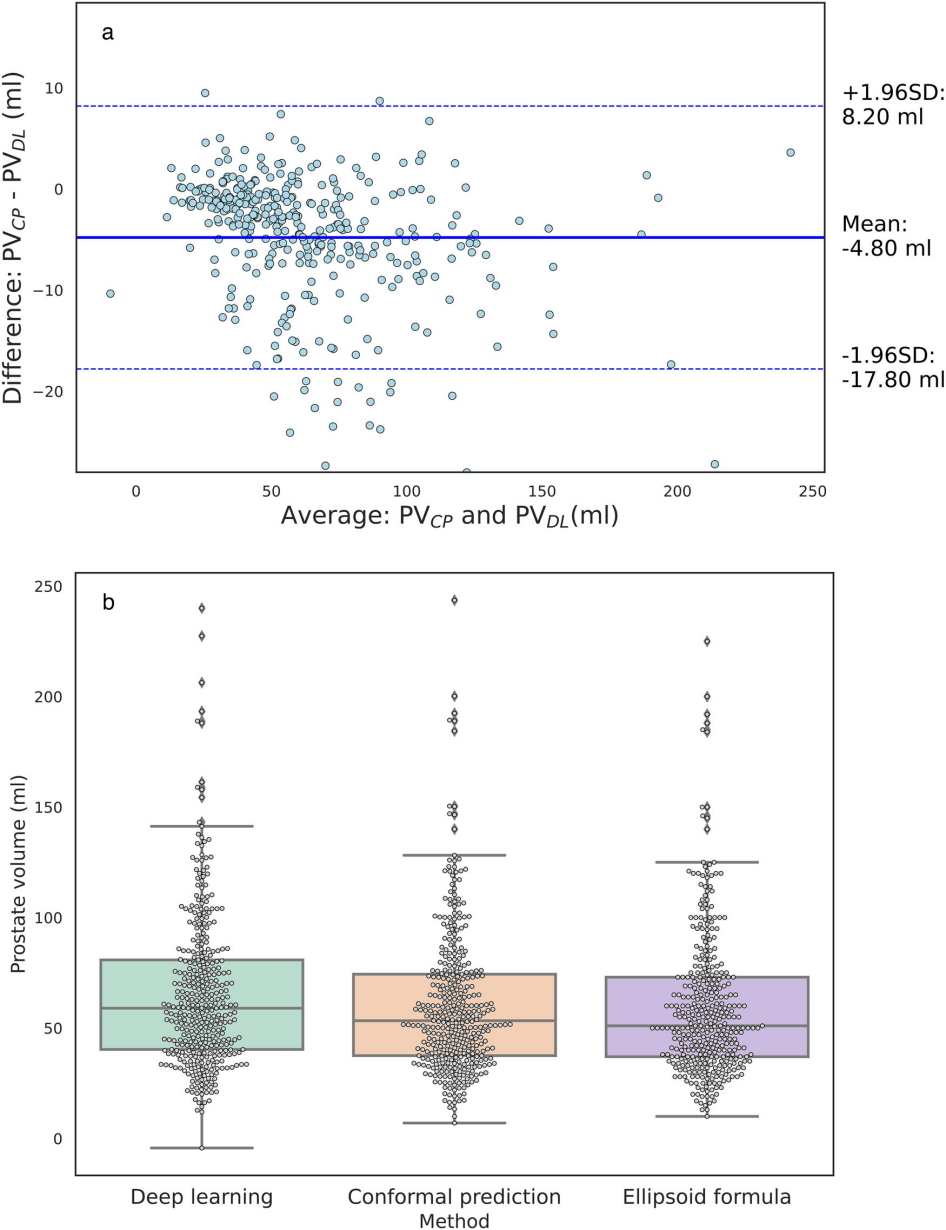
**a** Bland–Altman plot comparing prostate volumes measured by the deep-learning algorithm ${{{\rm{PV}}}}_{{DL}}$ and by the conformal predictor ${{{\rm{PV}}}}_{{CP}}$. The solid lines represent the mean difference and the dashed lines the limits of the agreements, calculated as a mean difference $\pm$ 1.96 SD. **b** Boxplot depicting the distribution of calculated prostate volumes by the deep-learning algorithm, conformal predictor and ellipsoid formula, respectively. Conformal prediction was applied with a confidence level of 85%

**Fig. 6:**
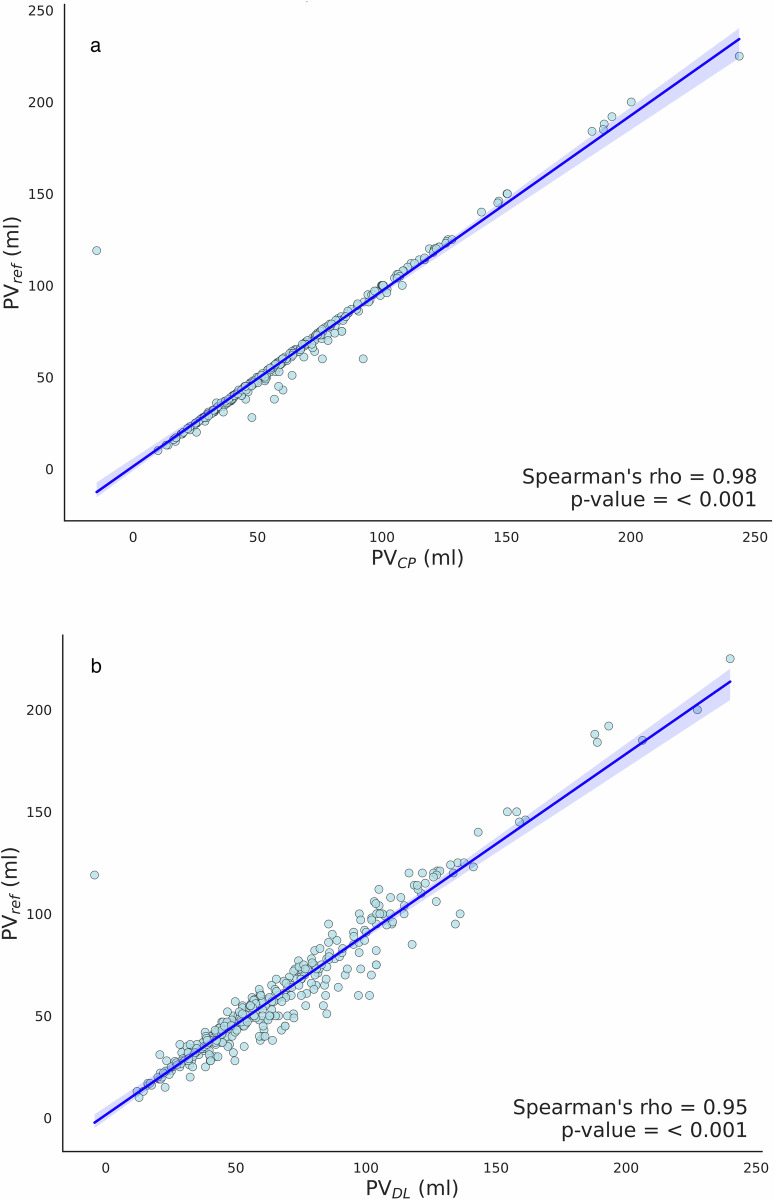
**a** Spearman correlation analysis for the prostate volume measured by the PI-RADS 2.1 ellipsoid formula ${{{\rm{PV}}}}_{{ref}}$ and by the conformal predictor ${{{\rm{PV}}}}_{{CP}}$. **b** Spearman correlation analysis between the prostate volume from the PI-RADS 2.1 ellipsoid formula ${{{\rm{PV}}}}_{{ref}}$ and by the deep-learning algorithm ${{{\rm{PV}}}}_{{DL}}$. Highlighted regions correspond to the 95% confidence interval calculated through bootstrapping. Conformal prediction was applied with a confidence level of 85%

Inter-agreement between ${{{\rm{PV}}}}_{{DL}}$ and ${{{\rm{PV}}}}_{{CP}}$ is shown in Fig. 5a, where we observe a systematic tendency of the DL to calculate larger ${{{\rm{PV}}}}_{{DL}}$ without CP (mean difference (95% limits of agreement) ${{{\rm{PV}}}}_{{CP}}$ vs. ${{{\rm{PV}}}}_{{DL}}$: − 4.80 mL (− 17.80 mL; 8.20 mL)). The box and whisker plots in Fig. 5b and Table 2 show the mean and standard deviation for all compared volumes. Figure 6 shows the results of the Spearman correlation analysis, where both measures presented a significant positive correlation with ${{{\rm{PV}}}}_{{ref}}$. A larger positive correlation was observed for ${{{\rm{PV}}}}_{{CP}}$ (0.98).

## Discussion

This retrospective multi-center study investigated the impact of estimating uncertainty in a DL-based prostate segmentation algorithm using CP in the PV calculation. Our results showed that first, CP significantly improved the prostate segmentation quality of a 2D nnU-Net DL model as measured by the DSC and ASD. Second, the predicted probabilities obtained from the DL model were calibrated significantly better after applying CP. Third, CP significantly improved accuracy in PV assessment as indicated by a reduced RVD and a stronger agreement via Bland–Altman plots and ICC compared to the reference standard. Our results support the hypothesis that CP can significantly improve the accuracy of DL-based PV assessment by relying on reliable pixel predictions with a pre-defined degree of certainty from the DL segmentation model.

Alongside our primary analysis using an alpha of 0.15—matching the 85% inter-observer agreement among radiologists—we also evaluated performance across alpha values (0.01, 0.05, 0.10, and 0.20). Lower alpha values (0.01, 0.05, and 0.10) caused under-segmentation, underestimating PV, and overestimating PSAd, which can adversely affect clinical decisions regarding PC risk stratification [2, 3]. In contrast, higher alpha values (0.20) led to over-segmentation, potentially underestimating PSAd and obscuring cancer risk. However, over-segmentation might be preferred in radiomics analysis, where the area surrounding the region of interest is often critical [27, 28]. In our study, an alpha of 0.15 balanced, excluding unreliable predictions with expert performance, aiding reliable PV estimation in clinical workflows [5, 14].

Various methods have been proposed to automate prostate segmentation and PV calculation [14, 25]. Meglič et al and Cuocolo et al trained U-Net model variants on cohorts from public and private datasets, achieving DSCs of 84 to 90% and RVDs of − 8.84 to 13.17%. While not directly comparable, our results suggest that our DL segmentation model performance falls within these ranges [14, 25]. Unlike previous studies [5, 14, 25], our approach incorporates a UQ mechanism through Mondrian ICP, with results that suggest that our approach might outperform others in prostate segmentation and PV assessment [5, 14, 25].

In terms of alternative CP methods, Venn predictors and cross-conformal prediction share theoretical roots with Mondrian ICP but face limitations in prostate segmentation and PV assessment [29]. Venn predictors are designed for multi-class scenarios, adding unnecessary complexity to binary tasks like prostate segmentation [30]. While cross-conformal enhances uncertainty estimation using multiple calibration sets, it requires repeated model retraining, which can be impractical for high-dimensional MRI data where rapid processing is essential [19].

In contrast, alternatives that do not belong to the CP family, such as probabilistic U-Net and Monte Carlo dropout, rely on specific output distribution assumptions [21, 22]. These methods also require substantial computational resources, and calibrating their uncertainty estimates can be challenging [30]. Such factors can prolong inference times and lead to unreliable estimates, affecting clinical applicability [21, 22, 29]. Mondrian ICP, however, provides reliable uncertainty estimates without substantial computational overhead, making it well-suited for this application. Its model-agnostic nature allows for easy integration into various segmentation architectures, enhancing usability [19, 29, 30].

Previous studies have identified systematic differences in PV calculation when evaluating DL models in scenarios resembling clinical deployment with heterogeneous data [4, 10, 14]. These differences might hinder the application of the DL models in clinical practice [4]. From this perspective, the Mondrian ICP only accepts predictions meeting a specified confidence level, flagging unreliable predictions for further inspection while providing a PV assessment based on reliable ones. This approach can increase synergy between physicians and the DL system, enabling the DL system to report dependable PV measures. This is reflected in our PV agreement results, where CP significantly increases the agreement of the measures when compared to the DL model without a UQ mechanism. Moreover, the multi-center and multi-vendor evaluation data suggest that Mondrian ICP might increase the robustness of the DL algorithm against heterogeneous data sources.

This study has several limitations. First, it is retrospective; prospective studies are needed to confirm causality. Second, we employed a simple conformal predictor; more advanced versions might yield better results. Third, we limited the study to CP’s capabilities to flag unreliable pixel predictions. Future research could explore the interaction between physicians and CP outputs for enhanced human-DL interaction. Additionally, our reliance on ensemble predictions, while common, may introduce blurring that could affect CP’s effectiveness. Examining different ensemble techniques on PV calculations could provide valuable insights. Further, incorporating a broader range of metrics in future evaluations could deepen understanding of CP’s effects on PV assessment and prostate segmentation accuracy. Finally, CP may require re-calibration when applied to new datasets, as shifts in data distribution could impact its predictive validity. Establishing systematic re-calibration protocols would be crucial for maintaining the accuracy and reliability of the algorithm.

## Supplementary information

ELECTRONIC SUPPLEMENTARY MATERIAL

## Data Availability

The datasets used and/or analyzed during the current study are available from the corresponding author (E-mail: alvaro.f.quilez@uis.no) on reasonable request.

## Abbreviations

ASD: Average surface distance
BS: Brier score
CP: Conformal prediction
DL: Deep learning
DSC: Dice score coefficient
ECE: Expected calibration error
ICC: Intraclass correlation coefficient
ICP: Inductive conformal prediction
PC: Prostate cancer
PSA: Prostate-specific antigen
PSAd: Prostate-specific antigen density
PV: Prostate volume
RVD: Relative volume difference
UQ: Uncertainty quantification
