# Supplementary material for: Impact of uncertainty quantification through conformal prediction on volume assessment from deep learning-based MRI prostate segmentation
Source: Insights Imaging. 2024 Nov 29;15:286. doi: 10.1186/s13244-024-01863-w (PMC11607187; doi:10.1186/s13244-024-01863-w)
Supplement: Supplementary file 1 — ELECTRONIC SUPPLEMENTARY MATERIAL [file 13244_2024_1863_MOESM1_ESM.pdf]

Impact of uncertainty quantification through conformal prediction on volume assessment from deep learning-based MRI prostate segmentation
ELECTRONIC SUPPLEMENTARY MATERIAL

Value	PV and segmentation assessment (N=377)					
	DL algorithm without CP			DL algorithm with CP		
	≤35 ml	>35ml < 50 ml	≥50 ml	≤35 ml	>35ml < 50 ml	≥50 ml
DSC (%)	77.49 ± 10.18	78.89 ± 8.71	82.53 ± 8.02	93.24 ± 8.47	92.10 ± 6.98	94.74 ± 6.92
ASD (mm)	0.71 (0.57and 1.54)	0.84 (0.55 and 1.81)	0.75 (0.54 and 1.38)	0.08 (0.04 and 0.19)	0.10 (0.04 and 0.25)	0.08 (0.03 and 0.19)
**RVD (%)**	7.20 ± 12.06	9.71 ± 11.07	7.64 ± 11.41	3.34 ± 8.36	4.05 ± 7.74	2.07 ± 9.42
PV (ml)	29.59 ± 7.11	46.32 ± 6.71	84.96 ± 32.36	28.51 ± 6.55	43.89 ± 5.31	80.82 ± 31.85

Table S1 Effect of uncertainty quantification through conformal prediction in deep learning-based prostate volume assessment and prostate segmentation, stratified by prostate volumes. Higher values for DSC are better, whilst values closer to 0 for RVD and ASD are better. Conformal prediction was applied with a confidence level of 85%.

Value	Calibration (N=377)					
	DL algorithm without CP			DL algorithm with CP		
	≤35 ml	>35ml < 50 ml	≥50 ml	≤35 ml	>35ml <50 ml	≥50 ml
ECE (%)	0.65 ± 0.21	0.68 ± 0.27	0.77 ± 0.30	0.10 ± 0.03	0.13 ± 0.02	0.12 ± 0.05
BS (%)	0.17 ± 0.08	0.21 ± 0.10	0.26 ± 0.22	0.03 ± 0.04	0.05 ± 0.03	0.05 ± 0.13

Table S2. Effect of uncertainty quantification through conformal prediction in the calibration of the deep learning-based prostate segmentation, stratified by prostate volumes. Lower values for ECE and BS are better. Conformal prediction was applied with a confidence level of 85%.

Supplementary Figures

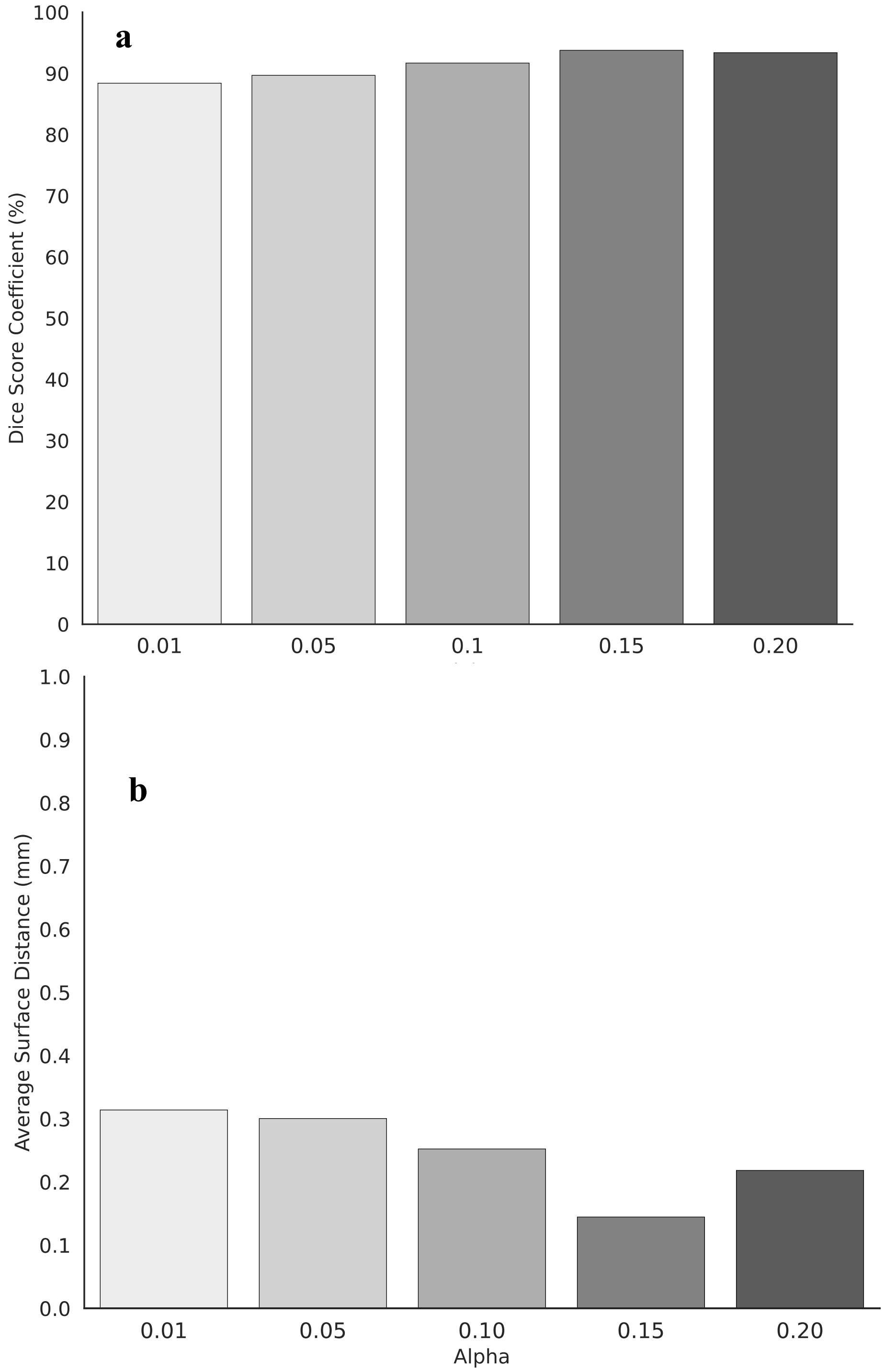

Figure S1. (a) Bar plot depicting the effect of alpha level on the average Dice Score Coefficient (DSC, %) when applying conformal prediction**. (b)** Bar plot depicting the effect of alpha level on the average Average Surface Distance (ASD, mm) when applying conformal prediction. Higher values for DSC are better, whilst values closer to 0 for ASD are better.

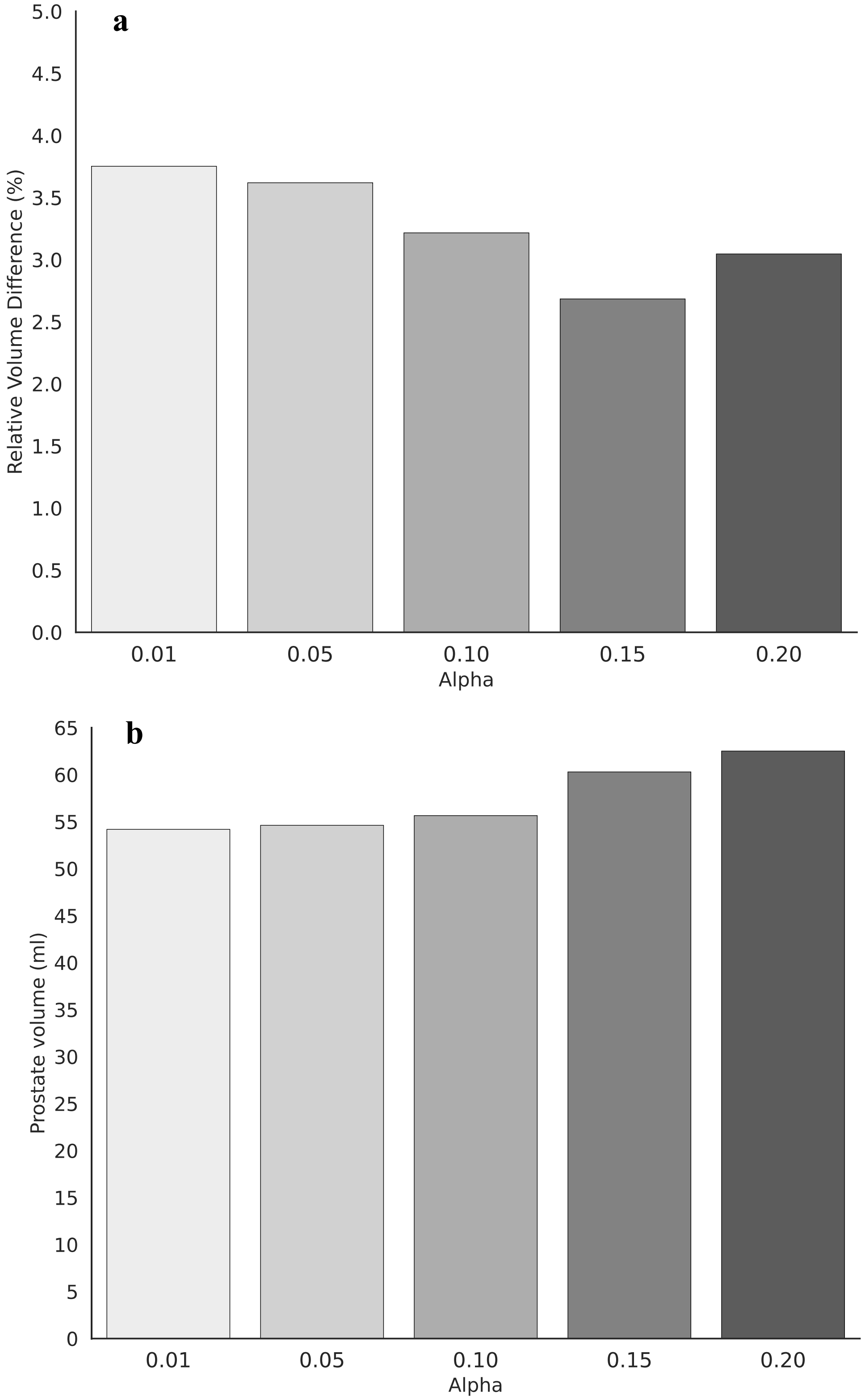

Figure S2. (a) Bar plot depicting the effect of alpha level on the average Relative Volume Difference (RVD, %) when applying conformal prediction**. (b)** Bar plot depicting the effect of alpha level on the average Prostate Volume (PV, ml) when applying conformal prediction. Values closer to 0 for RVD are better, whilst PV values closer to the ground truth PV calculated with the ellipsoid formula (59.43 ml) are better.
